# Developing a Rational, Optimized Product of *Centella asiatica* for Examination in Clinical Trials: Real World Challenges

**DOI:** 10.3389/fnut.2021.799137

**Published:** 2022-01-14

**Authors:** Kirsten M. Wright, Janis McFerrin, Armando Alcázar Magaña, Joanne Roberts, Maya Caruso, Doris Kretzschmar, Jan F. Stevens, Claudia S. Maier, Joseph F. Quinn, Amala Soumyanath

**Affiliations:** ^1^Department of Neurology, Oregon Health & Science University, Portland, OR, United States; ^2^Oregon's Wild Harvest, Redmond, OR, United States; ^3^Department of Chemistry, Oregon State University, Corvallis, OR, United States; ^4^Department of Pharmaceutical Sciences, Oregon State University, Corvallis, OR, United States; ^5^Linus Pauling Institute, Oregon State University, Corvallis, OR, United States; ^6^Oregon Institute of Occupational Health Sciences, Oregon Health & Science University, Portland, OR, United States; ^7^Department of Neurology, Veterans Affairs Portland Health Care System Center, Portland, OR, United States

**Keywords:** placebo, translation, *Centella asiatica*, botanical, dietary supplement, reproducible, clinical trials

## Abstract

Botanical products are frequently sold as dietary supplements and their use by the public is increasing in popularity. However, scientific evaluation of their medicinal benefits presents unique challenges due to their chemical complexity, inherent variability, and the involvement of multiple active components and biological targets. Translation away from preclinical models, and developing an optimized, reproducible botanical product for use in clinical trials, presents particular challenges for phytotherapeutic agents compared to single chemical entities. Common deficiencies noted in clinical trials of botanical products include limited characterization of the product tested, inadequate placebo control, and lack of rationale for the type of product tested, dose used, outcome measures or even the study population. Our group has focused on the botanical *Centella asiatica* due to its reputation for enhancing cognition in Eastern traditional medicine systems. Our preclinical studies on a *Centella asiatica* water extract (CAW) and its bioactive components strongly support its potential as a phytotherapeutic agent for cognitive decline in aging and Alzheimer's disease through influences on antioxidant response, mitochondrial activity, and synaptic density. Here we describe our robust, scientific approach toward developing a rational phytotherapeutic product based on *Centella asiatica* for human investigation, addressing multiple factors to optimize its valid clinical evaluation. Specific aspects covered include approaches to identifying an optimal dose range for clinical assessment, design and composition of a dosage form and matching placebo, sourcing appropriate botanical raw material for product manufacture (including the evaluation of active compounds and contaminants), and up-scaling of laboratory extraction methods to available current Good Manufacturing Practice (cGMP) certified industrial facilities. We also address the process of obtaining regulatory approvals to proceed with clinical trials. Our study highlights the complexity of translational research on botanicals and the importance of identifying active compounds and developing sound analytical and bioanalytical methods for their determination in botanical materials and biological samples. Recent Phase I pharmacokinetic studies of our *Centella asiatica* product in humans (NCT03929250, NCT03937908) have highlighted additional challenges associated with designing botanical bioavailability studies, including specific dietary considerations that need to be considered.

## Introduction

Botanical products are widely used by the public for their reputed health care benefits. Consumers in the United States (US) spent over $10 billion on herbal supplements in 2020, a record-breaking 17.3% increase from 2019 (1). The popularity of these products arises from familiarity with their folk medicine uses, vigorous commercial advertising, and their ready availability for self-selection through retail outlets. The US Food and Drug Administration (FDA) allows the marketing of botanical products as “dietary supplements” under the 1994 Dietary Supplements Health and Education Act (DSHEA). Notably, under DSHEA botanical products may be marketed without proof of efficacy, but must comply with labeling requirements limiting and qualifying the claims that are made. Nevertheless, consumers take these products with an expectation of a particular pharmacological effect or health benefit. At the same time, the FDA does provide for the development and registration of herbal products as “botanical drugs” for which proof of efficacy is a requirement (2).

While there is a significant body of literature on preclinical studies performed on botanicals, relatively few of these materials have been evaluated in formal, rigorous clinical trials. Additionally, several recent large-scale trials have failed to demonstrate a clinical effect of the botanical product under study (3). Recent articles led by authors at the National Institutes of Health (NIH) Office of Dietary Supplements (ODS) and National Center for Complementary and Integrative Health (NCCIH) have highlighted the particular challenges involved in the clinical evaluation of botanical supplements (3–5). Significant factors include the natural variation in plant materials and the multiplicity of available products of a given botanical. Similar challenges, and the need for standardization, have been noted for translational studies and clinical evaluation of plant foods that may promote health (6–8).

Important guidelines for the conduct of valid clinical evaluation of botanicals have been outlined (3–5). For the product these include: using a preparation method that closely matches traditional use or one that was used in supporting preclinical studies, confirmation that the active compounds of the botanical are present and remain stable throughout the trial period, selecting a dose expected to deliver therapeutic levels of the active compounds, formulating a product that is palatable and acceptable to study participants, and creating a matching placebo for successful blinding. The participants, treatment duration, and study end points selected must also be appropriate for the expected effects. It is now recommended that prior to performing costly efficacy trials, initial studies confirming bioavailability of the active compounds from the trial product, as well as identifying biological signatures in response to the intervention demonstrating relevant, mechanism-related target engagement are needed (3). Ultimately the health benefits of a botanical intervention need to be verified experimentally in efficacy trials (7). Given the inherent variability in raw botanicals and their products, it is acknowledged that it will not be possible to ensure total consistency between products tested in different trials. However, the provision of sufficient chemical analytical information and deposition of voucher samples would allow for comparison between the products used in separate studies (4, 5).

Here we present the process of designing a product made from *Centella asiatica* (L.) Urban (family Apiaceae), following the guidelines described above, for use in clinical trials relating to its potential use in the amelioration of cognitive decline. *Centella asiatica* (CA) is a small, perennial, creeper that grows in swampy areas of tropical and subtropical regions of Asia and Africa including Madagascar and Seychelles (9–11). The medicinal uses of CA can be traced from early documentation by the Indian physician Sushruta (*ca*. 1200 BC), to present worldwide use in commercial topical and oral products for skin and gastrointestinal conditions (12–14). Of particular relevance to our group's work is CA's importance in Ayurvedic medicine as a “medhya-rasayana” herb (i.e., one that has rejuvenating effects, boosts memory, prevents cognitive deficits, and improves brain function) (15–17). In the West, CA and CA dietary supplements sold under its Sri Lankan name “gotu kola” are marketed for its reputed benefits on brain and nerve function. Common preparations include tinctures (hydroethanolic extracts) or capsules containing powdered CA herb or a dried CA extract. A search for “*Centella asiatica*” and “gotu kola” on the NIH ODS labels database yields 657 and 1,476 hits, respectively (18), suggesting that there are around 1,500 dietary supplements containing CA currently available in the US.

The neurotropic and neuroprotective effects of CA have been widely studied and documented (14, 19). The vast majority of studies in the literature report data from preclinical models; however several small clinical studies also report CA's ability to improve memory, mood, or brain function in different population groups, including children (20), young adults (21, 22), middle-aged adults (23), or older adults (24–28). A meta-analysis failed to find a positive effect of CA on cognition (29); but as noted previously, the trials reviewed varied widely in the CA product tested, the level of product details provided, the subject population, the end points examined, and the quality of the methodology (19, 30) making direct comparisons between studies difficult.

Our group has been studying the mechanisms and active compounds associated with the cognitive effects of a hot water extract of CA, “CAW.” We have reported that CAW at doses of 200 to 1,000 mg/kg/day administered in the drinking water improves cognitive function in the Tg2576 (31) and 5xFAD (32, 33) mouse models of Alzheimer's Disease (AD), and also in aged wild-type (WT) mice (34–36). These effects are associated with improved antioxidant responses, mitochondrial activity, and synaptic density in the mouse brain (32, 34, 36, 37) and *in vitro* in neuroblastoma cells (38) and/or mouse primary neurons (39, 40).

**Figure 1:**
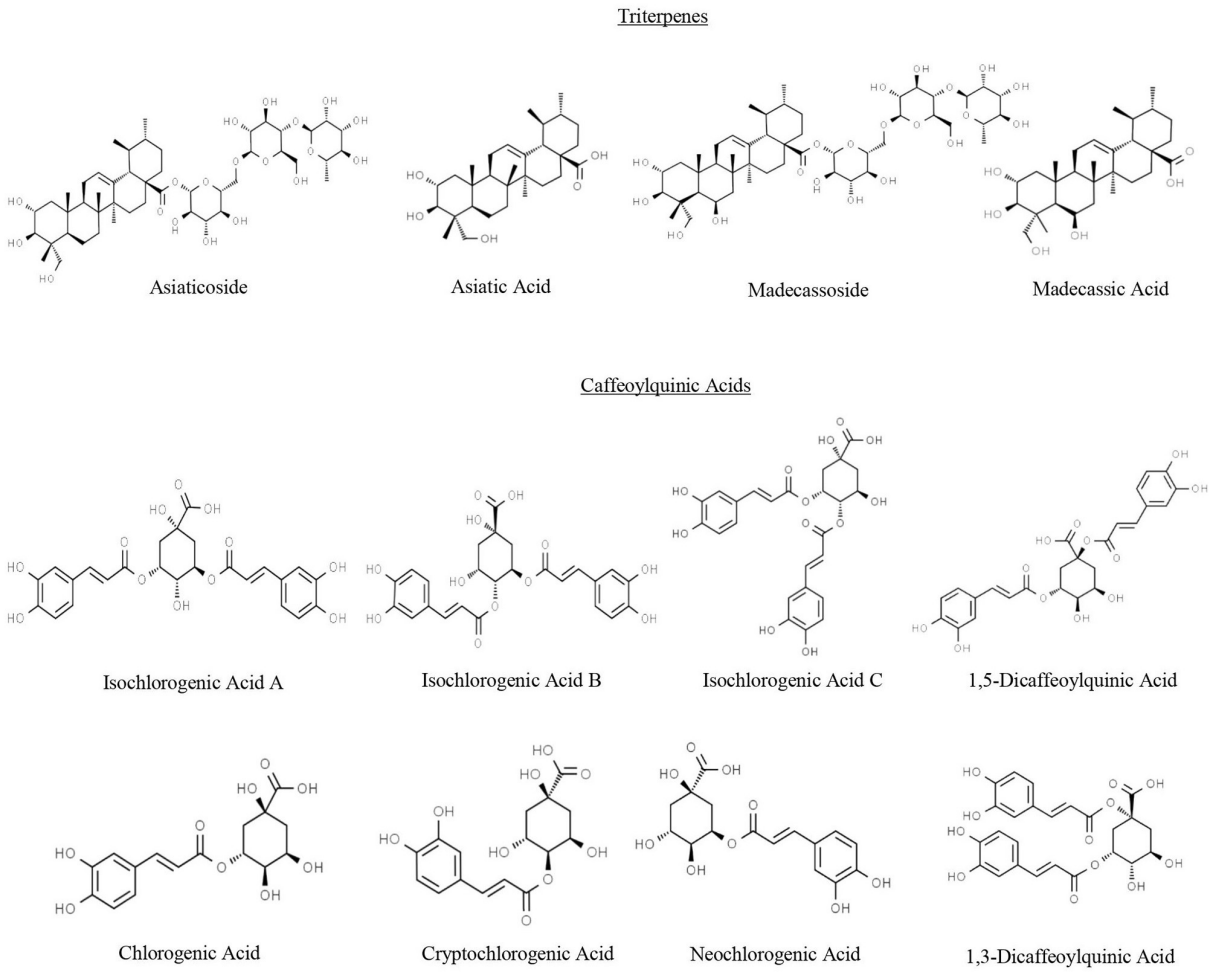
Chemical structures of triterpenes and caffeoylquinic acids found in *Centella asiatica*. Structures were obtained from Chemspider.

CA's biological activity has classically been ascribed to its characteristic triterpene (TT) compounds (Figure 1), chiefly asiatic acid (AA) and madecassic acid (MA), and their glycosides asiaticoside (AS) and madecassoside (MS), respectively (12). The International Union of Pure and Applied Chemistry's (IUPAC) names for these compounds are available on PubChem (41). The neurotropic and neuroprotective effects of these TT compounds, particularly AA and AS, are well-documented (19); however, in our preclinical studies, we have found that another group of specialized compounds in CA, mono- and di-caffeoylquinic acids (mono- and di-CQAs), also contribute to CA's neurological effects (19). The nomenclature of these compounds in the literature is inconsistent and IUPAC names are provided in a recent review (42). CAW and equivalent concentrations of di-CQAs (but not TTs) were found to protect neuroblastoma cells from beta amyloid (Aβ) toxicity *in vitro* (43) and improve antioxidant and mitochondrial gene expression in these cells (38). A CAW-equivalent mixture of mono and di-CQAs improved cognition *in vivo* in the 5xFAD mouse model of AD (44). CAW, as well as some TT and CQA compounds in isolation, reverse Aβ related loss of dendritic arborization and spines in mouse primary hippocampal neurons (40). These data led us to conclude that both TT and CQA content would be important to evaluate, optimize, and document for any future clinical trial CA interventional products.

Based on these preclinical studies, CAW appeared to be a good candidate for development as a “botanical drug” for the treatment of cognitive decline, in both normal and pathological aging, notably AD. Since CAW elicits cognitive improvement in aged, but not young, wild-type mice (34), and in mouse models of AD (31–33), an appropriate target population for a botanical drug made from CAW was deemed to be older subjects (age 65 years and over) experiencing cognitive decline in normal or pathological aging.

Due to the unavailability of a commercially prepared product that matched the composition of CAW used in our preclinical trials, and a company willing to comply with FDA reporting requirements, we elected to develop a custom product containing CAW for use in clinical trials. This product will be referred to as *Centella asiatica* product (CAP).

Here we describe our approach to developing CAP including how we encountered and addressed the challenges that arose, in particular those typically associated with botanical products.

## Materials and Methods

### Product Manufacture

#### Dosage Calculation and Delivery Method

Human doses equivalent to the mouse doses used in our preclinical studies (200–1,000 mg/kg/d) were estimated by interspecies (allometric) scaling (45). An oral delivery method was selected to mimic the preclinical studies. Additional consideration was placed on long-term compliance and convenience of consuming the dosage form in the target population selected.

#### Raw Material Selection: Identification, Chemical Characterization, and Evaluation of Contaminants

##### Sourcing of Material

To produce ample investigational product for the translational studies, including stability, bioavailability, safety, and biological signature evaluation, a source of a large quantity of single-batch raw CA herb material was identified. The form of the plant used in traditional herbal medicine (and our preclinical studies) is the dried aerial tops of CA, usually obtained from cultivated sources, so focus was placed on sources of aerial material to maintain ethnobotanical relevance and clinical applicability. Efforts were made to obtain organic material if possible (to minimize exposure to environmental toxins) and plant material that had been dried but had not undergone any other known processing apart from milling. Trade samples (80 g) of six different commercial sources of dried CA aerial parts, raw material (designated CA-1, CA-2, CA-3, CA-6, CA-7 and CA-8) were obtained through the dietary supplements company Oregon's Wild Harvest (Redmond, OR) and underwent quality control (QC) and chemical fingerprinting prior to purchasing a large quantity of raw material for product manufacture.

##### Identity Tests

First, organoleptic tests to confirm characteristic features (visual appearance, plant part, smell, and taste) and Fourier-transform infrared spectroscopy (FTIR) were performed on each of the six trade samples by the quality control laboratory at the Oregon's Wild Harvest. For organoleptic analysis, focus was placed on confirming the following characteristics: greenish-brown color with tan pieces, leafy, sour, a bit mint-like flavor, and a leafy aroma. FTIR spectroscopy is an established chemometric method for the identification of botanical powders where the spectrum of obtained samples is compared to a composite spectrum of multiple previous batches of the same botanical (46, 47). These crucial cost-effective analyses eliminated false or grossly adulterated products before proceeding with more costly chemical characterization and product manufacture.

To confirm identity of the botanical materials as CA, ethanolic (CAE) and aqueous (CAW) extracts were prepared from each trade sample using previously described methods (48). Extracts were analyzed using thin layer chromatography (TLC) alongside extracts prepared from CA materials used in our preclinical studies (CA-4 and CA-5) and commercial reference standards (Chromadex, Irvine, CA; Sigma Aldrich, St Louis, MO; TransMIT, Gießen, Germany) of the TT and CQA compounds shown in Figure 1. For TLC, 25 μL of each CAW (10 mg/mL) or CAE (10 mg/mL) extract was spotted onto a silica gel stationary phase (200 μm) plate with fluorescence indicator (particle size 20 μm) on aluminum backing (Lot #3110; Sigma Aldrich Z193291) alongside reference standards (1 mg /mL, 25 μL). The mobile phases used were ethylacetate: formic acid: glacial acetic acid: water (100:5:5:5) for separating the CQA's, chloroform: methanol: glacial acetic acid (90:10:5) to separate the TT aglycones (AA and MA), and chloroform: methanol: glacial acetic acid (75:25:5) to separate the TT glycosides (AS and MS). Visualization of zones was achieved using ultraviolet light (254 and 365 nm) for the CQAs or 1% vanillin/sulfuric acid spray followed by heating for the TTs (49).

##### Chemical Characterization

For chemical characterization, high-pressure liquid chromatography coupled to high-resolution mass spectrometry (LC-HRMS) (50) was performed on the trade samples and the CA materials used in preclinical studies to identify and quantify the TT and CQA compounds of interest. Untargeted LC-HRMS of the raw plant materials was also performed. Analytical data on mass, retention time, and peak area of all components detected using both positive and negative electrospray ionization (ESI) were recorded as “fingerprints” of the CA extracts to create an archival record of each material.

##### Evaluation of Contaminants

To assess the presence of contaminants, the Safe Quality Food Institute (SQFI)-certified quality control laboratories of Oregon's Wild Harvest performed impurity analysis of the sourced CA trade materials. Microbial content (aerobic plate count, yeast, mold, *Escherichia coli*, total coliforms, and *Salmonella*) was determined using the company's standard plating methods (Binary Detection Technology; BDT, AOAC 2005.03 and AOAC 2013.01). Aflatoxin analysis was not required due to the absence of mold in any of the samples. Heavy metal content (lead, arsenic, cadmium) was determined at Oregon's Wild Harvest using atomic absorption spectroscopy, while mercury and pesticide analyses were performed by a third-party contract laboratory (Columbia Food Laboratories, Portland, OR) using inductively coupled plasma mass spectrometry and gas chromatography coupled to mass spectrometry, respectively. In addition to the raw plant material, heavy metal and pesticide content was also determined in water extracts prepared from these plant materials, as the levels could either be enhanced (for water soluble contaminants) or reduced (for water insoluble contaminants) by water extraction.

##### Evaluation of Bulk Materials Selected for Manufacture

Identity tests and contaminant evaluation were repeated at Oregon's Wild Harvest on the bulk samples chosen for use in product manufacture, with attention to the use of standard Oregon's Wild Harvest sampling procedures (United States Pharmacopeia (USP) #40; Part 561; Articles of Botanical Origin). In addition, analysis of aflatoxins was performed out of an abundance of caution, even in the absence of mold. Aflatoxin analysis was performed either at Oregon's Wild Harvest (Agri-Screen, Neogen Co.; United States Department of Agriculture (USDA)-Grain Inspection, Packers and Stockards Administration (GIPSA) 2010-006) or by a third-party contract laboratory (Romer Labs, Union, MO) using high-performance liquid chromatography (HPLC). Chemical characterization by LC-HRMS (as in section Chemical Characterization) was also performed on these bulk materials.

#### Selection of Manufacturing Facilities and Formulation Development

##### Identification of Certified Facilities

It was essential to perform the product manufacture in facilities with Current Good Manufacturing Practice (cGMP) certification. In addition, such facilities needed to be capable of large-scale water extraction of CA and drying of the extract. Written communications were sent to numerous cGMP certified facilities followed up by phone calls which identified one suitable facility (Ashland Laboratories, previously Pharmachem Laboratories (PCL); Kearny, NJ). Additional facilities were identified (Oregon's Wild Harvest) that could complete product manufacture including blending of the dried extract with excipients and final product labeling and packaging.

##### Formulation of the Clinical Trial Product and Matching Placebo

In designing the clinical trial product formulation, delivery method, palatability and placebo matching were considered. Due to the oral delivery method selected for the target population, the sticky, hygroscopic nature of the dried CAW, and available facilities for mass manufacture, it was decided to dry the CAW extract onto a suitable matrix to optimize further blending and packaging steps, and aid its efficient dispersion in water for consumption by trial participants. The percent loading of CAW onto the matrix was determined based upon the final product's properties and the amount of matrix determined to be safe for consumption (51). Essentially different ratios of CAW and matrix were mixed in water, dried by lyophilization, and examined for homogeneity, texture, and water dispersibility.

Additional excipients were identified to improve palatability and color matching for a placebo. The recommended maximum daily intake, normal amount or percentage in food, normal daily intake, caloric content, and median lethal dose (LD50) of each excipient were used to identify potential excipients and determine safety and dosing. This information was found using the FDA Code of Federal Regulations, the National Library of Medicine ToxNet Toxicology Data Network, the Global Safety Management, Inc. Safety Data Sheet, the USDA and United States Department of Health and Human Services 2015-2020 Dietary Guidelines for Americans, the certificate of analysis and data sheet provided by the supplier of each excipient, and available journal articles discussing safety and tolerability of each specific excipient.

Each potential excipient evaluated was analyzed independently by the investigators at the Oregon Health & Science University's Bioanalytical Shared Resource/Pharmacokinetics Core Lab (Portland, OR) for TT and CQA content using HPLC-tandem mass spectrometry (HPLC-MS/MS) and excluded from consideration if any TT or CQA were identified as present. For the detection of TTs, selected reaction monitoring was performed on an Applied Biosystems Q-Trap 4000 LC-MS instrument (Framingham, MA). Chromatographic separation was achieved using a Poroshell 120 EC18 column (3 mm id × 50 mm; 2.7 μ), Poroshell ultra high-performance liquid chromatography (UHPLC) guard column, and a methanol:ammonium acetate gradient (Santa Clara, CA). Triterpenes were detected as their ammonium adducts with positive ion mode electrospray ionization using the following MS/MS transitions (m/z): AA (506/453), MA (522/451), AS (976/453; 976/635), MS (992/487; 992/451). Two internal standards were used with chrysin being detected as the molecular ion (255/255) and ursolic acid as its ammonium adduct (474/411; 474/191). For the detection of CQAs and their associated metabolites, HPLC-MS/MS was performed on an Applied Biosystems 5500 QTRAP HPLC-MS instrument (Framingham, MA) using an analytical method modified from that described by Nair et al. (52). Chromatographic separation was achieved using a C8 reversed-phase column (Agilent Zorbax Eclipse plus C8 Rapid resolution 4.6 × 150 mm 3.5 μ; Santa Clara, CA), an Agilent Zorbax Eclipse plus C8 Rapid resolution guard column (4.6 × 12.5 mm 5 μ; Santa Clara, CA), and an acidified acetonitrile:water gradient. CQAs, their metabolites, and internal standards were detected using negative ion mode electrospray ionization and the following MS/MS transitions (m/z): mono-CQAs (353/191); di-CQAs (515/353; 515/191); caffeic acid (179/135); ferulic acid and isoferulic acid (193/134); dihydrocaffeic acid (181/109); ^13^C_9_-caffeic acid (188/143); ^13^C_3_-ferulic acid (196/136); d_3_-isoferulic acid (196/134); and d_3_-dihydro-isoferulic acid (192/135).

Excipients with any reported neurological activity were eliminated from consideration. A total of 19 excipients were evaluated (14 for palatability, 3 for coloring, and 2 as a carrier matrix for dispersability).

#### Manufacturing Process, Analysis of Intermediates, and Final Product

##### Extraction and Drying

Selected CA herb batches (CA-3 and CA-6) were purchased in bulk by Oregon's Wild Harvest and shipped from the two original suppliers directly to the identified extraction and drying facility (Ashland Laboratories). Using one of three stainless steel jacketed reactors with stainless steel agitators each with water-cooled condensers and receivers (2 at 500-gallon capacity, 1 at 750-gallon capacity) and an evaporator pfaudler (3' diameter x 8' high; glass lined), CA dried herb was extracted using distilled water under reflux (160 g herb: 2 L water to match the laboratory scale method used in our preclinical studies). The hot extract was cooled from 212 to 150°F, filtered through a 200 mesh screen, then filtered through filter paper to remove particulates, and a 3 L aliquot (free of spray drying carrier matrix) was removed and sent to OHSU for lyophilization. This sample was used for phytochemical and percent loading analyses.

The remainder of the filtered extract was spray dried onto a carrier matrix at a target 66% loading capacity using a Type III A-No. 7 304 gas fired stainless steel APV anhydro spray dryer with centrifugal atomizer, Gaulin Homogenizer feed pump, with Clean in Place (CIP) system. Due to size limitations of the extraction equipment, two separate extractions were performed to obtain the required total amount of dried extract. In the first process, a mixture of CA-3 (14.50 kg) and CA-6 (30.5 kg) was extracted with 562.5 L of hot water and spray dried onto a carrier matrix (Batch 1). In the second extraction, CA-6 (45 kg) was extracted with 562.5 L of hot water and spray dried onto the same carrier matrix (Batch 2). A 200 g sample of spray dried CAW from each batch was sent to Oregon State University for analysis, while the bulk of the spray dried product was shipped to Oregon's Wild Harvest for quality control, blending into the drug product, packaging, and storage.

##### Analysis of Intermediates

Intermediates in the manufacturing process were evaluated for CAW content based on the concentrations of TTs and CQAs. LC-HRMS data was used to calculate the amount of CAW loaded onto the carrier matrix by comparing relative concentrations of the active compounds (mg/g material) in the Batch 1 and Batch 2 lyophilized samples and their respective spray dried counterparts. For this, the mean loading was calculated from values for all the individual TT and CQA compounds except for AA and MA. The data suggested that AA and MA levels were disproportionately skewed by possible hydrolysis of the more abundant glycosides, AS and MS, during spray drying.

##### Product Blending and Packaging

Based upon the amount of TTs and CQAs determined to be present in each batch of spray dried material, and the quantity of spray dried product (here referred to as “Gotu Kola Extract Preblend”) needed for QC, stability studies, and the proposed trials, Batch 1 spray dried material was blended with Batch 2 spray dried material. The quantity from each batch used in the blend were determined using the desired phytochemical content of the final product.

Because the weights of “Gotu Kola Extract Preblend” corresponding to the different doses of intervention and the placebo would contain different amounts of matrix, additional matrix material was added to the placebo and to the lower CAW dose to equalize the matrix content across all 3 doses. All other excipients (for color and flavor) were added in equal amounts to each dose. The required excipients/additional matrix for each dose of CAP were blended in bulk in quantities sufficient for 200 individual dose sachets per dose. The correct weight and composition of blended excipients for a single dose of CAP was weighed into individual sachets (stand-up metalized barrier pouches, Item 183-60, Associated Bag Company, Sparks, NV) at Oregon's Wild Harvest. The required weight of “Gotu Kola Extract Preblend” was hand weighed and added to each individual sachet, to give the required dose of CAW. The filled sachets were heat sealed. All sachets were stored in the freezer (−20°C) until their use for quality control, stability tests, or dispensing for use in the proposed clinical studies.

##### Quality Control

All final CAP doses including the placebo were analyzed using TLC to confirm TLC zone profiles characteristic of CAW and to confirm the presence of TTs and CQAs in CAP containing CAW 2 g or 4 g but their absence in placebo. LC-HRMS was used to quantify TT and CQA in CAP 2 g and 4 g and to be able to identify those peaks in the untargeted fingerprint of CAP that belong to the excipients rather than CAW. All products were also analyzed for microbial, pesticide and heavy metal content. Uniformity of weight of the sachets was checked by weighing each sachet to confirm the correct weight for that dose and minimal variation between packages of a given dose.

### Product Stability

To comply with regulatory requirements for obtaining Investigational New Drug (IND) status for a manufactured botanical product, an accelerated stability study was conducted in collaboration with the Food Innovation Center (FIC; Oregon State University, Portland, OR). Multiple sachets of each dose of CAP were placed at two different accelerated storage conditions (25 ± 2°C/60% ± 5% relative humidity and 40 ± 2°C/75% ± 5% relative humidity) for 32 days. In addition, to compare routine, commonly available storage conditions, sachets were placed at −20°C (freezer), 4°C (refrigerator), and ambient temperature (benchtop) for the same duration. All samples were transferred to −20°C after 32 days until analysis by LC-HRMS (*n* = 6 replicates of each dose for −20°C; and *n* = 3 replicates of each dose for other temperatures). The placebo was not assessed for stability since the end point was analysis of levels of TT and CQAs. For each known bioactive compound, the mean level in the samples stored for 32 days under the other 4 conditions were compared to the level seen in the samples stored at −20°C (deemed to be the most stable) to determine degradation. Visual and olfactory inspections were also performed on all samples.

### Bioassay to Confirm Biological Activity

An *in vivo* bioassay appropriate to the antioxidant and cognitive enhancing effects of CAW was used to confirm biological activity of the final formulated product. *Drosophila melanogaster* fruit flies with a mutation in the *sniffer* gene *sni1* allele were determined to be an appropriate model due to an impaired locomotion phenotype consistent with neurodegeneration from oxidative stress (53). Study vials (35 mL, 24.5 × 95 mm) were prepared using standard *Drosophila* food with the addition of CAW (10 mg/mL), CAP (equivalent to 10 mg/mL CAW), excipients alone (equivalent to those in CAP), or deionized water (control). Flies (males and females) were placed in vials containing either control food or experimental food for 7 days before testing. Fast phototaxis assays were conducted as a measure of locomotion and cognitive function as previously described (54, 55) using a countercurrent apparatus (56) and a single light source. Data was analyzed using GraphPad (v.5 for Windows, San Diego, CA) and one-way ANOVA with a Dunnett's post-test to determine significance to the untreated control or to flies treated with the excipients alone.

### Regulatory Considerations

Early in the planning of the proposed translational work, exploration was conducted into the necessity of Investigational New Drug (IND) status from the FDA. Written communication was sent to a member of the FDA's Botanical Review Team and, subsequently, an enquiry to the Food, Dietary Supplements and Cosmetics IND Jurisdiction Team (FIJT) was submitted. Following a recommended pre-IND consultation, an IND application including a detailed Chemical and Manufacturing Controls (CMC) document was submitted as per the FDA's published guidance document (2). For documentation of prior human use and human safety, online databases such as the ODS Labels Database (18), and the FDA and CFSAN Adverse Event Reporting System (FAERS and CAERS) databases were used to illustrate widespread use of CA preparations with minimum toxicity. Several previous clinical trials that had been done without notable adverse effects were included; and the use of CA as an edible plant, and its safety when brewed into a tea (similar to CAW preparation), were highlighted. Published animal toxicity studies of CA preparations were also included. Additional approvals for proposed studies were obtained from the clinical trial sponsor (NCCIH) and the OHSU Institutional Review Board (IRB) following receipt of IND status.

## Results

### Product Manufacture

#### Dosage Calculation and Delivery Method

Human doses equivalent to the mouse doses (200–1,000 mg/kg/d) used in our preclinical studies were estimated to be 2–10 g CAW per day using interspecies scaling (45). Since robust cognitive improvements in mice had previously been observed at doses of 200 mg/kg/d (31, 32, 34, 36) and 500 mg/kg/d (33), a 10 g/day dose in humans was determined to be unnecessary. For the planned Phase I studies, the dose range was adjusted to 2 g and 4 g of CAW per day based on the highest reported, well-tolerated, human dose of CA triterpenes (240 mg/day) (57). The standard maximum content of a capsule or tablet is 500 mg. Daily doses of 2 g and 4 g CAW translated to 4–8 capsules per day, which was considered unsustainable and inconvenient for an elderly population. Therefore, it was decided to provide the CAW as a powder to be dispersed in one glass (10–12 ounces) of water and consumed orally, closely mimicking the administration of CAW in the preclinical studies. This delivery format required the design of a new formulation with the addition of excipients to improve dispersion and palatability, and coloring agents to mask the placebo.

#### Raw Material Selection: Identification, Chemical Characterization, and Evaluation of Contaminants

##### Sourcing of Material

It was challenging to identify sources of a single batch of CA material that was available in the required large quantity (90 kg). All suppliers were willing to provide a trade sample but could not guarantee that the larger amount would still be available after preliminary evaluations had been completed. There was variability in the form of bulk raw materials available including powder, finely milled “tea cut,” coarsely milled “cut and sift,” and dried whole plant materials.

##### Identity Tests

Most of the available plant material was a cut or milled version of the dried herb, making formal identification and verification of authenticity through examination against botanical keys not possible. However, all the trade materials evaluated passed organoleptic, FTIR and TLC criteria for identification as CA. Specifically, FTIR provided acceptable matches to prior database spectra and TLC confirmed the presence of TTs and CQAs characteristic of CA (data not presented). The TLC zone profiles were essentially similar, qualitatively, to the preclinical study voucher samples, although quantitative variations in band intensity between the materials were noted.

##### Chemical Characterization

Targeted analysis was used to evaluate the content of TTs and CQAs in the trade samples, with the goal of identifying material containing within ±10% of active compounds found in CA-4 and CA-5 that had shown biological activity in prior preclinical studies (31–34, 36, 38–40, 43). There was high variability between CA accessions in the content (% w/w in CAW) of total TTs (0.5–11%), total mono-CQAs (0.1–0.4%) and total di-CQAs (0.1–1.4%), as well as in individual members of each class, identified by LC-HRMS (50). Interestingly, the TTs and CQAs did not vary in the same way (i.e., plant materials that had higher levels of TTs did not necessarily also have higher levels of CQAs). No single CA trade sample matched CA-4 or CA-5 (50). Validation of the LC-HRMS method, results of targeted analysis of TTs and CQAs in samples CA-1 to CA-8 and a comparison of their fingerprints using principal component analysis have been published (50). Detailed LC-HRMS data from untargeted analysis of the eight samples has been archived.

##### Evaluation of Contaminants

All trade materials passed the following acceptance criteria for microbial content established at Oregon's Wild Harvest for their raw materials and products: aerobic plate count <10,000,000 colony forming units (cfu)/g; mold or yeast <100,000 cfu/g; coliforms report cfu/g; *E. coli* <10 most probable number (mpn)/g; *Salmonella* absent in 25 g (Supplementary Table 1). Other analyses revealed that even those trade samples that had been certified “organic,” had detectable levels of pesticides, and most also contained heavy metals (Table 1). Generally, heavy metal and pesticide concentrations were lower in the extracts than the starting material, but two pesticides (quizalofop and 2,4-D) appeared more prominently in the extracts than the raw materials from which the extracts originated.

**Table 1:** Heavy metal and pesticide content of five CA trade samples acquired for possible use in preparing CAP, and their water extracts.

**Source**	**Cadmium (ppm)**	**Lead (ppm)**	**Arsenic (ppm)**	**# Pesticide residues**	**Highest pesticide residue (mg/kg)**
CA-1 Raw	<0.1	1.8	<0.5	Not measured	Not measured
CA-2 Raw	0.3	0.3	<0.5	11	2.5 (Chlorpyifos)
CA-2 Extract	<0.1	<0.2	<0.5	5	1.2 (Quizalofop)
CA-3 Raw	1.2	1.6	<0.5	4	0.059 (Propiconazole)
CA-3 Extract	0.435	0.455	<0.5	1	0.018 (Propiconazole)
CA-6 Raw	0.6	2.5	<0.5	6	0.049 (2,4-D)
CA-6 Extract	0.233	0.358	<0.5	2	0.064 (2,4-D)
CA-7 Raw	5.9	4.3	<0.5	8	0.044 (Carbendazim)
CA-7 Extract	2.444	0.766	<0.5	1	0.073 (2,4-D)
CA-8 Raw	1	4.1	<0.5	None detected	None detected

##### Evaluation of Bulk Materials Selected for Manufacture

Based upon their phytochemical profile (50) and relatively low contaminant content, CA-3 and CA-6 were selected for the manufacture of CAP. Interestingly, CA-3 was certified organic while CA-6 was not but was listed as not genetically modified (non-GMO). CA-6 had unusually high levels, and CA-3 had unusually low levels, of AS compared to CA-4 and CA-5. The decision was made to mix the two plant materials to yield an extract with TT levels closer to those found CA-4 and CA-5. Bulk quantities of these materials were purchased and analyzed for potential impurities (Table 2) and phytochemical profile to confirm that they matched their respective trade samples. Both CA-3 and CA-6 raw materials passed the standards for microbial content (Supplementary Table 1) and aflatoxins (absent) and had phytochemical profiles matching their trade sample.

**Table 2:** Contaminant content of bulk CA samples selected for mass manufacture.

**Plant** **materials**	**Microbial levels**	**Heavy metals (ppm)**	**Pesticides (mg/kg)**
CA-3	Conforms to Oregon's Wild Harvest specifications	Lead (2.088) Arsenic (<0.5) Cadmium 1.13	Diphenylamine (0.028) Propiconazole (0.038)
CA-6	Conforms to Oregon's Wild Harvest specifications	Lead (2.434) Arsenic (<0.5) Cadmium 0.537	Cypermethrin (0.018) 2,4-D (0.039) Hexachlorobenzene (0.010) Permethrin (0.033)

For heavy metals, tolerances were obtained from the United States Pharmacopoeia (USP). The USP #40; Part 561; Articles of Botanical Origin suggests the following guidelines for residual levels of heavy metals: cadmium 0.5 μg/g, lead 5 μg/g, arsenic 2 μg/g. Although the CA-3 material did not conform to this specification, the levels of cadmium reduced following water extraction (Table 1), allowing for the continued progression with using this material. When evaluating the acceptability of the pesticide residue levels, measured levels were compared to maximum residue limits (MRL) available in the USP #40; Part 561; Articles of Botanical Origin and the European Pharmacopeia 8.2 (Ph.Eur; section 2.8.13). Cypermethrin, hexachlorobenzene and permethrin were below the USP and Ph.Eur limits of 1, 0.1 and 1 mg/kg, respectively, however MRL values were not available for diphenylamine, 2,4-D, and propiconazole.

#### Selection of Manufacturing Facilities and Formulation Development

##### Identification of Certified Facilities

Three facilities with current Good Manufacturing Practice certification (cGMP) were identified that were capable of large-scale water extraction of CA and/or drying the large volume of extract to be generated. Most companies identified in initial searches performed hydroalcoholic but not aqueous extractions and were thus eliminated. Only one facility identified had both capabilities (Ashland Laboratories); however, the method for drying offered was spray drying onto a matrix and not freeze-drying. A trial batch did not show appreciable degradation of TT and CQA following spray drying compared to a lyophilized counterpart (data not shown), so it was decided to contract out the CAW production and drying to Ashland Laboratories. cGMP and SQFI-certified facilities at Oregon's Wild Harvest were identified to be used for final product manufacture (blending of the dried extract and excipients) and packaging.

##### Formulation of the Clinical Trial Product and Matching Placebo

The three doses of CAP were designed to differ only in CAW content, i.e., 0 g (placebo) or 2 g and 4 g of CAW, but contain identical amounts of excipients, including the spray drying matrix substance. This would ensure any measured biological activity in a placebo-controlled study is solely attributable to CAW. It was determined that the spray dried product could contain up to 66% CAW (i.e., a 2:1 ratio of CAW to matrix) and this was the target loading aimed for during manufacture, based on the theoretical weight of CAW extracted.

**Figure 2:**
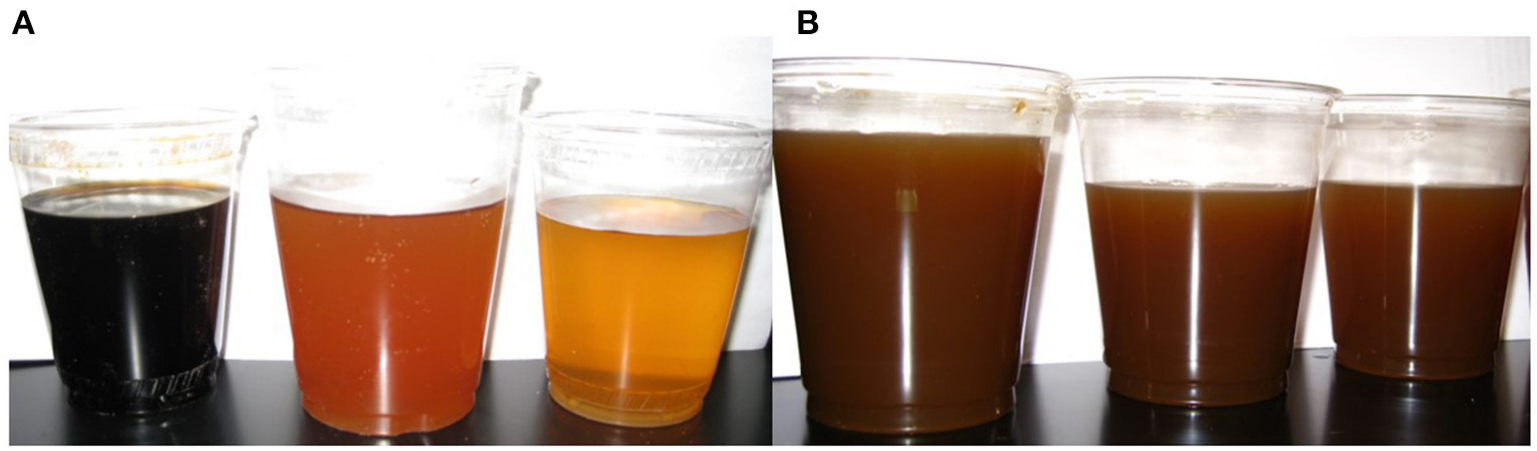
Coloring excipient test and final CAP matching evaluation. **(A)** Three different coloring agents were dissolved in 8 oz. of water and compared against one another for color matching with CAW. **(B)** 0, 2, and 4 g CAP, containing the selected coloring agent and additional excipients for flavor matching, dissolved in 8 oz. of warm water.

Of the 19 excipients evaluated for taste, color, neurological activity, and phytochemical content, six were eliminated due to TT and/or CQA content and two were reported to have cognitive enhancing properties. Six other excipients were eliminated due to challenges with color matching (Figure 2A). In the end, five excipients were selected with one being a food grade colorant, three for palatability, and one as the carrier matrix. The identities of these excipients are not disclosed in this publication due to intellectual property considerations but can be provided under agreement. The three doses of CAP, when reconstituted in water, are shown in Figure 2B and were not readily distinguishable by color, smell, or taste.

#### Manufacturing Process, Analysis of Intermediates, and Final Product

##### Extraction and Drying

CA-3 and CA-6 were extracted and dried in two separate batches. Batch 1 (CA-3 plus CA-6) was expected to have a lower concentration of AS than Batch 2 (CA-6 only) due to the presence of CA-3 plant material which had low levels of this compound (50). The spray dried product from Batch 1 (10.85 kg) was blended with spray dried product from Batch 2 (6.40 kg) at Oregon's Wild Harvest to yield “Gotu Kola Extract Preblend” (17.25 kg). The quantities were based on the calculated total amount of material needed for QC, stability studies and the proposed clinical trials, and utilized the maximum amount of Batch 1 available to ensure a lower AS level similar to CA-4 and CA-5.

##### Analysis of Intermediates

Prior to incorporation into the drug product, further quality control tests were performed on “Gotu Kola Extract Preblend.” Samples of the lyophilized extract aliquots (without matrix materials) from the two batches were compared by TLC with their spray dried counterparts, “Gotu Kola Extract Preblend,” and CA-4. The compounds typical of CA in the test materials were identified in each of the samples. CA's TT and CQA compounds were analyzed in each manufacturing intermediate using LC-HRMS. Using untargeted LC-HRMS in negative and positive ionization mode, the retention time, peak area and high-resolution mass of “Gotu Kola Extract Preblend” was recorded as a fingerprint (data archived).

Despite the targeted loading value of 66%, the actual percent loading of CAW onto the matrix was below this value and differed between the two extraction batches. Batch 1 was calculated to have ~51% loading of CAW onto the matrix, while Batch 2 had just over 34% loading. By calculation from CAW content in the two spray dried batches, “Gotu Kola Extract Preblend” was estimated to have 7.73 kg CAW in 17.25 kg of material (about 45% loading). Based on this, the amounts of “Gotu Kola Extract Preblend” required for CAP 2 g CAW and CAP 4 g CAW were calculated as 4.4 g and 8.8 g, respectively.

##### Product Blending and Packaging

Individual sachets containing single doses of 0, 2, or 4 g of CAW and equal amounts of all the excipients (including the carrier matrix) were made and packaged as described in section Product Blending and Packaging.

##### Quality Control

**Figure 3:**
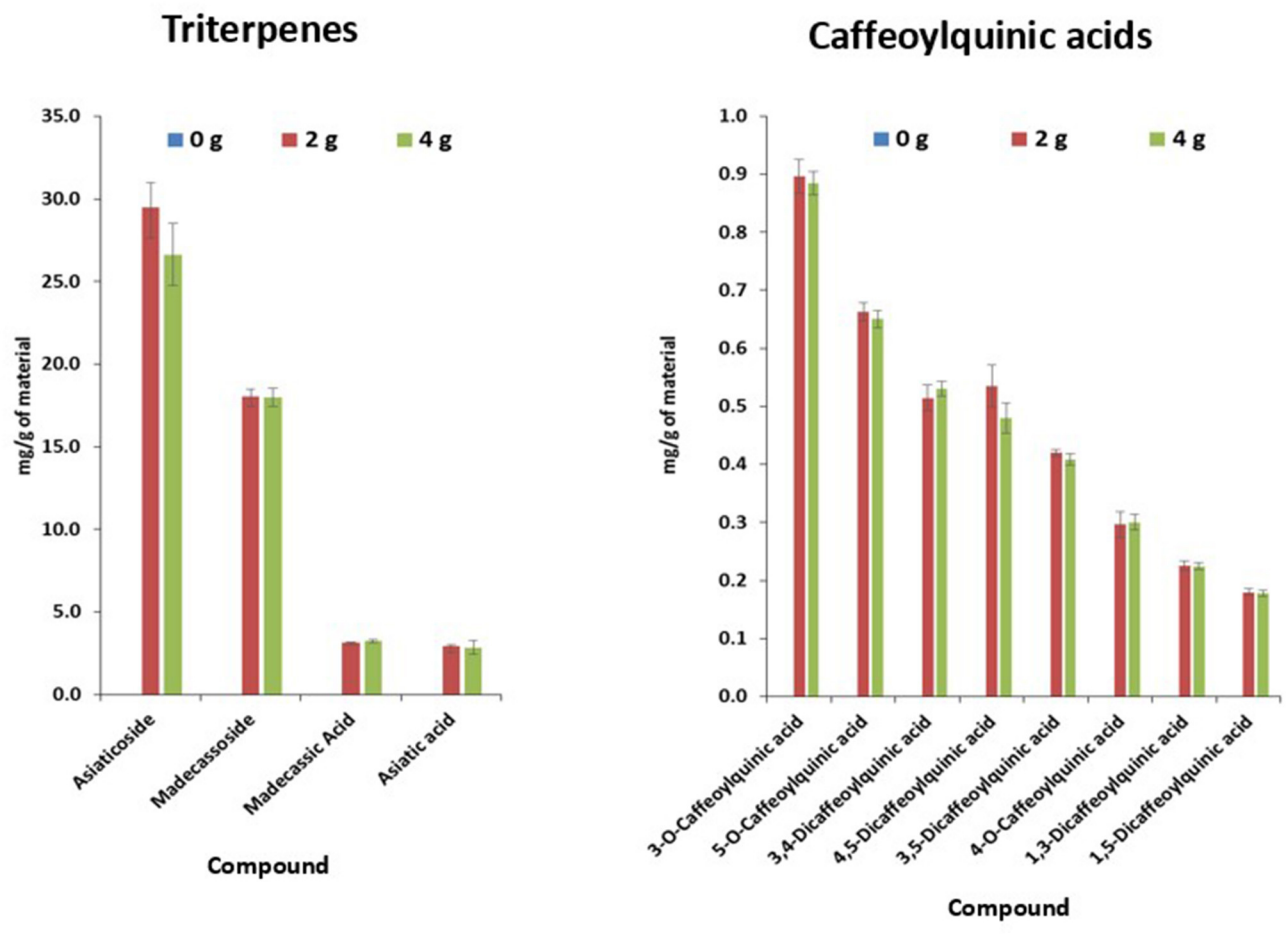
Concentration of triterpenes and caffeoylquinic acids found in 0, 2, and 4 g *Centella asiatica* product as determined by LC-HRMS. Sachets of the *Centella asiatica* water extract products (CAP; *n* = 5 per dose) were extracted with methanol and analyzed for the content of active compounds (triterpenes and caffeoylquinic acids) using LC-HRMS in positive and negative ion mode against commercial reference standards. The content of triterpenes and caffeoylquinic acids per gram of *Centella asiatica* extract was identical for the 2 g and 4 g doses of CAP and showed low variability indicating successful and uniform manufacture of the two doses. None of the specific analytes were detected in the 0 g dose, confirming their absence at detectable levels in the placebo which was comprised solely of the excipients used.

Product sachets passed Oregon's Wild Harvest criteria for uniformity of weight. Analysis of TT and CQA content of samples of the final CAP products, in comparison to the parent CAW extract, confirmed that the correct levels of CAW had been included in CAP 2 g and 4 g and that these compounds were absent in CAP 0 g (placebo). TT and CQA content in CAP 0, 2, and 4 g are shown in Figure 3. When contaminants were assessed, all three products remained within the limits for microbial levels (Supplementary Table 1). Heavy metal and pesticide content of all CAP doses are shown in Table 3. Cadmium and lead were detected at levels above the limit of quantitation in at least one of the CAP products. Of the various pesticides seen in the starting materials (CA-3 and CA-6) (Table 2), only two of them (diphenylamine and 2,4-D) were detected in the CAP (Table 3).

**Table 3:** Heavy metal and pesticide residue content in CAP 0, 2, and 4 g.

**Heavy metal or pesticide**	**CAP 0 g**		**CAP 2 g**		**CAP 4 g**	
	**Ppm (μg/g)**	**Amount per sachet (μg)**	**Ppm (μg/g)**	**Amount per sachet (μg)**	**Ppm (μg/g)**	**Amount per sachet (μg)**
Cadmium	<0.1 loq	(<1.1)	<0.1 loq	(<1.3)	0.26	3.81
Lead	<0.2 loq	(<2.2)	0.27	3.43	0.69	10.15
Arsenic	<0.5 loq	(<5.4)	<0.5 loq	(<6.4)	<0.5 loq	(<7.7)
Mercury	<0.01 loq	(<0.11)	<0.01 loq	(<0.13)	<0.01 loq	(<0.15)
Diphenylamine	<0.01 loq	(<0.11)	0.014	0.179	0.035	0.516
2,4-D	<0.01 loq	(<0.11)	0.024	0.306	0.024	0.354

*loq, limit of quantitation*.

### Product Stability

**Figure 4:**
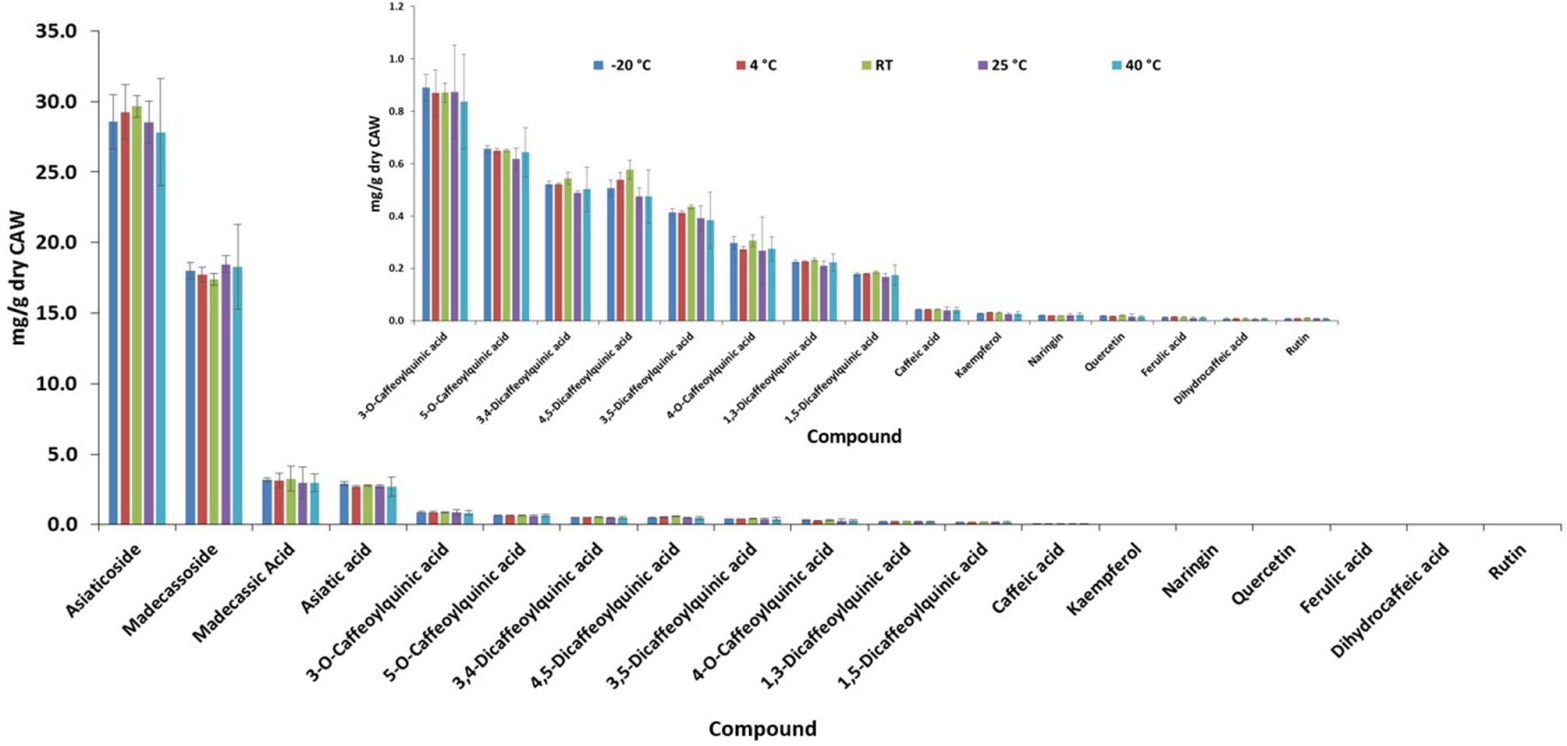
A 32-day stability test of CAP 2 g and 4 g showing unchanged levels of triterpene and caffeoylquinic acid components at all temperatures as determined by LC-HRMS. Storage conditions: 25 ± 2°C/60% ± 5% relative humidity, accelerated 40 ± 2°C/75% ± 5% relative humidity, fridge (4°C), ambient temperature (RT), and freezer (−20°C) (*n* = 3–6 per condition).

**Figure 5:**
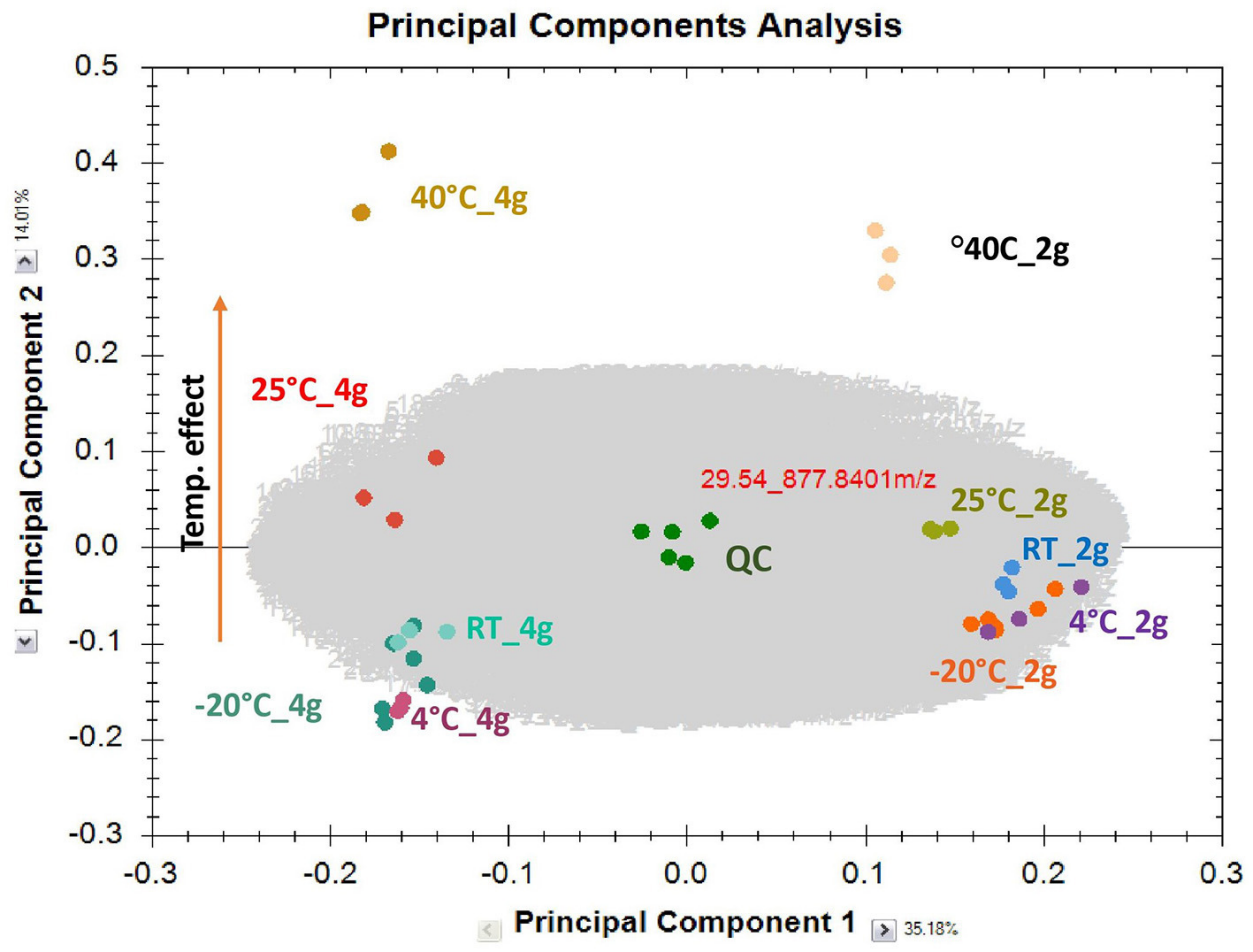
Principal Component Analysis (PCA) of 32-day stability test of CAP 2 g and CAP 4 g. Samples were stored in special chambers held at 25 ± 2°C/60% ± 5% relative humidity or accelerated 40 ± 2°C/75% ± 5% relative humidity, at ambient temperature (RT), in a refrigerator (4°C), or in a freezer (−20°C) (*n* = 3–6 per condition). Chemical fingerprinting analysis of CAP storage stability samples by untargeted data dependent acquisition was performed using LC-HRMS as described earlier (50). The content of each sachet was suspended in 70% v/v methanol (100 mL) containing formic acid (0.1% v/v). Samples were sonicated for 2 hrs with strong shaking every 30 min at room temperature. The suspension (1 mL) was centrifuged (15,000 rpm, 10 min) and diluted 100 times before injection. QC samples were obtained by pooling equal aliquots of each sample. Principal component analysis was performed in Progenesis QI software (V 2.4). All *m/z*-signals that triggered MS2 experiments (5,849) were used after log-transformation and Pareto scaling. The first principal component represents the maximum variation through the data. Next, another axis representing the next highest variation within the data is added orthogonal to the first one, and is designed as the second principal component. Each marker represents a sample; identically colored markers are replicate samples (*n* = 6, −20C; *n* = 5, QC; and *n* = 3 for all other groups). The PCA plot shows that samples that have a high degree of similarity in their chemical fingerprints cluster closely together. Clustering of the QC samples in the center of the plot confirms LC-MS platform stability. The shift from the left bottom quadrant to the upper left quadrant (4 g sachets) and from right bottom quadrant to right upper quadrant (2 g sachets) indicates that the chemical fingerprints are sensitive to storage temperature. Targeted analyses of the CAP stability samples conducted in parallel (Figure 4) indicated CQA and TT levels were unaffected by storage temperature; thus the observed shifts hint that either excipients or other components of CAW were sensitive to storage temperature.

Visual and olfactory inspection of the samples stored at different temperatures during stability analyses showed no obvious changes at lower temperatures; however, the samples stored in the 40°C accelerated condition showed some clumping. There was no difference in the levels of TTs and CQAs between the different storage conditions demonstrating stability under all conditions tested (Figure 4); however, principal component analysis (PCA) of all LC-HRMS peaks did show a temperature dependent separation of samples stored at the two accelerated storage conditions from those stored at −20°C, 4°C, and ambient temperature (Figure 5). Based on this data, it appears that CAP 2 g and CAP 4 g will be stable for at least 1 month if stored at −20°C, 4°C, or ambient temperature. While some changes were observed under accelerated storage conditions using PCA, the main active compounds appeared unaffected (Figure 4).

### Bioassay to Confirm Biological Activity

**Figure 6:**
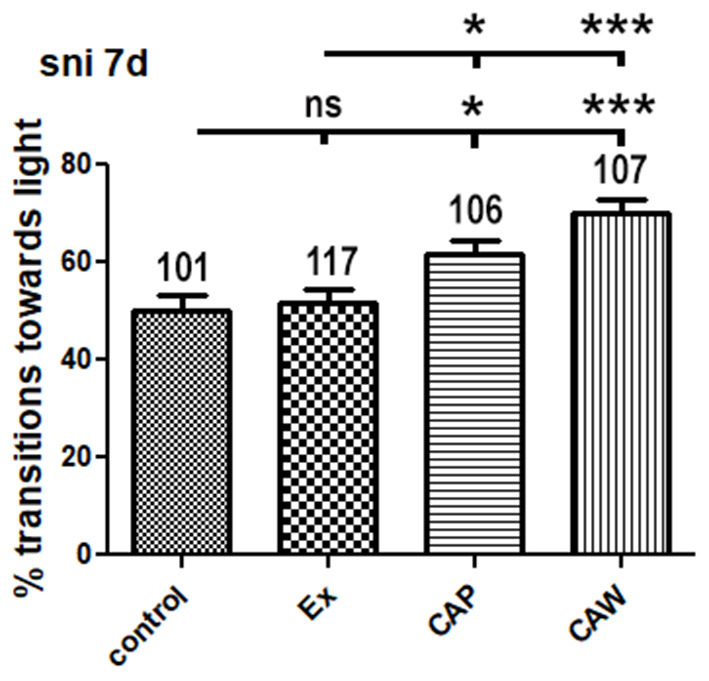
Percentage transitions toward light for *sniffer* flies treated with *Centella asiatica* water extract, *Centella asiatica* water extract product, excipients used in the manufacture of CAP or control food. *Drosophila melanogaster* fruit flies with a mutation in the *sniffer* gene *sni1* allele were fed standard food (control) or standard food supplemented with either *Centella asiatica* water extract (CAW; 10 mg/mL), *Centella asiatica* water extract product (CAP; equivalent to CAW 10 mg/mL), or the matching placebo for CAP containing only excipients (Ex; equivalent to the amount in CAP) for 7 days. Fast phototaxis was performed with flies (data from both sexes combined) and compared to either control or Ex treatment. The number of tested flies is given above the bars and the SEM is indicated. *p < 0.05, ***p < 0.001.

Using the fast phototaxis assay, there was no significant difference in transitions toward the light between the control and excipients-treated *sniffer* flies (Figure 6). CAW-treated flies showed significantly (*p* < 0.001) greater transitions toward light than control and excipients-treated flies. CAP treatment also significantly (*p* < 0.05) increased transitions toward light compared to control and excipients treatment. CAP and CAW treatment were not significantly different from each other. These data suggests that the formulated CAP product has similar neurological activity to the active component, CAW, and the excipients selected do not have appreciable neurological effects as measured in this model. This bioassay appears to be suitable for use in evaluating CAP biological activity in future studies where additional product manufacture will be necessary.

### Regulatory Considerations

The FIJT determined that an IND was required for two reasons (a) because the goal of our clinical studies was to develop CAW to mitigate a disease (cognitive decline) bringing it under the category of a drug rather than a dietary supplement, and (b) because CAW was not a currently lawfully marketed drug. After a few amendments to the initial submission, IND status for CAP was awarded. The success of the FDA IND application was pivotal to moving forward with the clinical study; both the study sponsor (NCCIH) and OHSU's IRB required IND status to be obtained before granting their respective approvals for clinical protocols.

## Discussion

### Chemical Variability and the Importance of Analytical Methods

Our experience with custom manufacture of a CA product for a clinical trial confirmed several of the earlier known challenges relating to botanical research. The first among these is the inherent chemical variability within a single botanical species. Levels of the bioactive TT compounds are reported to vary considerably between accessions of CA (19) and this was confirmed for both TTs and CQAs in the present study (50).

Identification of active compounds in a botanical, followed by chemical fingerprinting and quantification of known bioactives using validated methodology and authenticated reference standards, is an imperative step for rigorous translational studies. Such methods, as we have developed for CA; (50), can be used to identify (1) the material with the closest matching active compound profile to those used in preclinical studies showing biological activity, (2) variability between accessions from the same plant material, and (3) adulterated products. While it is valuable to apply targeted techniques to measure and control the content of known active compounds, plant raw materials and extracts also contain a vast number of other compounds, whose identity and/or contribution to the overall activity of the product may as yet be unknown. Therefore, the use of untargeted analysis to document a comprehensive range of analytical features (e.g., chromatographic retention time and mass spectral information) of both known and additional components is invaluable in preserving a complete picture of the chemical profile of a botanical product. The overall profile of different materials can be compared using principal component analysis for example (50). These analyses can, and should, be performed on the raw herb, prepared extracts, manufacturing intermediates, the final product(s) upon completion of manufacture, and the final product(s) during storage and use (e.g., in a clinical trial). We have taken this approach for our study.

The identification of sufficient, chemically similar raw material for all the planned studies is an important aspect in botanical translational medicine studies to ensure limited variability in phytochemical content and impurity profile. Ideally, a reliable, reproducible source of plant material with desirable and minimally variable chemical profile should be identified.

### Contaminants in Plant Materials

There are several unique environmental contaminants of concern when sourcing raw plant material for use in clinical trials. These include heavy metals, pesticides, microbes, mycotoxins, and polyaromatic hydrocarbons (PAH) (8, 58–60). While several pesticides are banned (57), it is more common that an upper limit of content is specified by regulatory authorities or pharmacopeias for specific pesticide residues, as well as for heavy metals, microbes, and mycotoxins. Limits for PAH content, while established for many food commodities, are still being considered for botanical products (60).

To assess the acceptability of the heavy metal content in the final CAP products (Table 3), we compared the content to published maximum daily intake recommendations. The American Herbal Products Association (AHPA) Guidance Policy (2012; current at the time of product manufacture) recommends the following maximum intakes: cadmium 4.1 μg/day, lead 6 μg/day, inorganic arsenic 10 μg/day, and methyl mercury 2 μg/day. The International Council for Harmonization of Technical Requirements for Pharmaceuticals for Human USE (ICH) Harmonized Guideline for elemental impurities (Q3D; 2014) lists the following values for permitted daily exposure (PDE) by the oral route: cadmium 5 μg/day, lead 5 μg/day, arsenic 15 μg/day, and mercury 30 μg/day. The amounts of cadmium, mercury, and arsenic, delivered per dose of CAP were less than the lower of the two recommended maximum values. However, the lead content in 4 g CAP (10.15 μg; Table 3) was higher than daily maximum intake of 5 μg/day for this metal recommended by both AHPA and the ICH. While this was concerning, the ICH guidelines (Q3D, section Bioassay to Confirm Biological Activity) mention that intermittent or short-term (30 days or less) dosing may be justification for allowing impurity levels higher than the established PDE. The FDA has allowed use of this product for a pharmacokinetic (PK) study where each participant only receives a single dose of CAP 4 g. However, a new batch of product with a more acceptable heavy metal profile may be needed for studies involving longer term dosing.

The Code of Federal Regulations (CFR; Part 180) lists maximum recommended level (MRL) values for pesticide residues in several food commodities. For example, for diphenylamine (CFR part 180.190) the tolerances in pears and apples range from 5 to 30 ppm. For 2,4-D (CFR part 180.142), the tolerances in various agricultural commodities range from 0.05 to 50 ppm. However, most botanicals used in dietary supplements are not included in these commodity lists. Information on allowable levels of these contaminants may be found in the USP and Dietary Supplements Compendium (DSC) sections on articles of botanical origin, and the European Pharmacopeia (Ph.Eur.), or guidelines from the ICH or World Health Organization (WHO) and Food and Agriculture Organization (FAO). Some limitations to finding permitted levels of pesticide residues in particular, are that not all pesticides are listed in the pharmacopeias. For example, maximum recommended level (MRL) values were not available in the USP, DSC or Ph.Eur. for diphenylamine and 2,4-D. Hence, it may be more relevant to consult documentation, where available, on maximum allowed daily intake rather than MRL. The FAO and WHO's Codex Alimentarus online pesticide database cites allowable daily intakes (ADI) of up to 0.08 and 0.01 mg/kg body weight for diphenylamine and 2,4-D, respectively, corresponding to 4.8 and 0.6 mg per day for a 60 kg adult. The National Science Foundation (NSF) International Standard/American National Standard organization has published maximum allowable levels per day (MAL) values for many pesticides (61). For diphenylamine, this value at 700 μg/day, is considerably lower than the FAO/WHO value, and at the time of our study, no MAL value was given for the pesticide 2,4-D. Both the residual levels (ppm) and the amount (μg) of diphenylamine and 2,4-D delivered by a single dose of CAP 2 g and CAP 4 g appear well below the available MRL (food commodities), ADI, and MAL values.

### Designing Botanical Interventions and Placebos for a Human Trial

The principal factors to consider in designing the product for our clinical trial were similarity to the material showing biological activity in preclinical studies, translation of the mouse doses to human studies, and the development of a suitable dosage form to deliver human doses along with a matching placebo. Our preclinical studies had examined a hot water extract of CA (CAW) that is produced by boiling CA in water under reflux for 2 h followed by filtration, freezing and lyophilization (31–38, 40, 42–44, 48). We were unable to find a commercial product that had been prepared in this way for use in the proposed human trials. We also considered using products made by a different extraction method (e.g., using ethanol), but which may have had similar levels of TT and CQAs. However, some companies that had such products expressed reluctance to provide information on their product to the FDA for an IND application. Consequently, we decided to use a custom-made hot water extract, and, with some difficulty, found a cGMP facility able to perform the extraction and drying. However, they were only able to provide a product spray-dried onto a carrier matrix rather than a lyophilized dried extract. This resulted in additional analytical studies to ensure the TT and CQA were not adversely affected by this process before proceeding with manufacture of CAP. In addition, it was observed that the percentage loading onto the carrier matrix during spray drying was significantly different from the target value of 66% and varied between batches (51 and 34%) despite efforts to maintain batch consistency. This introduces a need for further analytical measurements after drying of every batch to determine actual loading prior to final formulation and blending with excipients. Variability in loading must be monitored and controlled, if possible, to maintain consistent excipient levels between CAP batches. Otherwise, variations would need to be accounted for using batch specific placebo controls.

The human doses of CAW for our proposed study were selected by interspecies scaling (45, 62) and reference to earlier human studies (19) to limit the potential for toxicity at higher doses. The doses calculated were larger than the dose of CA or CA extract provided by most commercial CA products (around 500 mg/capsule). The larger dose (4 g) required that the product be administered as a liquid drink rather than swallowed as a capsule. We therefore had to formulate a product that was palatable and dispersed easily in water. This raised the issue of making a matching placebo, and some considerable effort was required to design one that had a similar taste and color to the CAW-containing product. It was also essential that any agents added to produce CAP were devoid of TT and CQA, safe for human use, and not known to have any neurological effects. It was important to make the placebo and CAW containing products identical in all respects except for CAW content, so that any differences in biological effects between CAW and placebo-treated groups could truly be ascribed to CAW.

### Regulatory Aspects

FDA IND status was required for CAW for the reasons mentioned earlier (section Regulatory Considerations). Although we were not required to provide extensive toxicology data on CA for the IND application, we did have to provide evidence of its likely safety. We cited widespread human use of CA as a dietary supplement and edible plant with limited reports of human toxicity, organ toxicity studies in animals, and the relative absence of adverse effects in clinical trials of CA. However, the relevance of this evidence to CAW was complicated by the large variability in the type of CA preparations that are used in dietary supplements, as well as in those used in earlier preclinical and clinical studies (19). For example, although previous studies had examined the effects of CA extracts on CYP drug metabolizing enzymes (63–67), the FDA recommended that we examine and report the drug interaction potential of our CAW water extract specifically (68). Also, since earlier preclinical studies on organ toxicity had been performed using other types of CA extracts (69–72), we obtained organ toxicity data from mice treated with CAW that could be used in the IND application. A point of caution regarding several reports of CA hepatotoxicity in humans (notably in review articles) (73–75)—an examination of the original papers cited showed that some preparations involved were multi-herbal products including CA where direct association could not be established.

### Looking Ahead to Clinical Studies

We have embarked on a series of translational studies with the ultimate goal of performing a Phase II trial examining the efficacy of CAW in ameliorating cognitive decline in humans. Important steps to optimize this future Phase II study would be to:

I. identify or develop a CAW product that matches the one used in our preclinical studies and contains an appropriate dose of TTs and CQAs likely to have a biological effect in humans;
II. confirm bioavailability of CA's active compounds by performing a plasma pharmacokinetics (PK) study following acute oral dosing of the CAW product;
III. determine safety and tolerability of the CAW product; and
IV. demonstrate target engagement by evaluating changes in specific biological signatures related to CAW's mechanisms of action that had been observed in preclinical studies.

In the present report, we describe our experience with Step I above. Steps II to IV would need to be performed in the selected target population (older adults of both sexes). Recently, CAP 2 g and 4 g have been used in two pharmacokinetic studies in older adults (NCT03929250, NCT03937908). The multiplicity of active compounds (TTs and CQAs) in CAW has required the development and validation of sensitive bioanalytical methodology to measure these CAW compounds in human plasma and urine at much lower concentrations than found in the plant extracts. For bioavailability studies from botanical extracts it is important to consider potential biotransformation of the phytochemicals during their transportation through the body. For example, in humans the TT glycosides of CA (AS and MS) appear to be hydrolyzed in the gut such that only the aglycones (AA and MA) appear in the plasma (57, 76–78). Similarly, the CQAs undergo extensive gut, Phase I and Phase II metabolism to metabolites such as caffeic acid, dihydrocaffeic acid, dihydroferulic acid, ferulic acid, isoferulic acid, dihydroisoferulic acid and hydroxyphenylpropionic acids, and their glucuronide and sulfate conjugates (79). The biodistribution of any these compounds may be relevant to the biological activity of CA and must therefore be evaluated.

The pharmacokinetic studies also required participants to observe a strict low phytochemical diet for 48-h prior to dosing and for 12 h post dosing. This is because of the ubiquitous distribution of CQAs in common foodstuffs and beverages, as well as the potential to obtain TTs from food sources, although the TTs have a more limited distribution in plants. Participants were instructed to avoid fruits, vegetables, nuts, spices, coffee, tea, whole grains, and whole wheat products for 48 hours prior to the pharmacokinetic studies. They were placed on an identical low phytochemical diet during each study visit to reduce dietary variability. They were asked to fast for 10 h prior to each visit and were not provided food for the first 2 h following CAP administration to account for differences in gastrointestinal transit time and dietary interference. While this may not be practical for studies involving prolonged consumption of CA, it is clearly needed for a pharmacokinetic study. Based on our preclinical *in vivo* studies reviewed earlier, we expect that 4–6 weeks of continuous administration of CAP will be needed to see evidence of target engagement in humans e.g., a reduction in oxidative stress markers or evidence of improved mitochondrial function and neuronal viability from brain imaging studies. Several months of CAP administration may be required to demonstrate a change in the rate of cognitive decline.

Some additional challenges are anticipated in upcoming dose escalation and safety studies. For the product, the success of blinding to placebo, and tolerability and acceptability of the product when consumed daily for extended periods of time are important parameters to evaluate. It is also important to monitor the stability of the product for the duration of the clinical trial whether stored by the investigators, research pharmacy, or the participants.

Here we present an example of a rigorous science-based approach to translating preclinical botanical studies to clinical trials. Matching the clinical trial material as closely as possible to that used in preclinical studies is important, as well as choosing a dose range for the product based on sound rationale (e.g., interspecies scaling). A valid, matched placebo must also be produced for comparison if appropriate to the study, and attention paid to the possible presence of confounding phytochemicals in the placebo materials or the diet of participants. Good botanical raw materials will contain adequate levels of active compounds and minimal contaminants. Sufficient material of this quality must be secured for the duration of the study to minimize variability in the test material throughout the study. Validated analytical methods specific to the botanical extract under consideration are essential and key to a successful clinical trial, from guiding the selection of raw materials, through product manufacture and validation of product stability during the trial. With the inherent variability of plant materials, the data from even the most carefully performed trial remains product specific. However, the careful documentation of product chemical characteristics will facilitate reproducibility between studies, as well as comparison of data from different trials.

### Significance of CAP Clinical Trials to the Use of CA in Traditional Medicines and Food

As well as its use as a traditional medicine, CA is an edible plant consumed regularly in the diet as a vegetable, juice, or tea in several Asian countries (80–84) and in South Africa (85, 86). Indeed CA's popularity in foods is increasing due to growing awareness of its health benefits (81). A question may arise regarding the relevance of clinical trials of CAP to the health benefits of traditional medicines or foods containing CA. Aside from the complicating issue of inherent variability of plant materials, the answer largely depends on how closely the product tested matches the preparation method, and amount of CA consumed. For cognitive benefits, in Ayurvedic medicine CA herb is reportedly prepared as the fresh juice “swarasam,” mixed in clarified butter as a “gritham,” or given in milk (15). In our preclinical studies, and in CAP, we used a hot water extract of CA (CAW). This extraction method was based, not on traditional preparation methods, but on earlier published studies demonstrating superior cognitive enhancing properties in rats of CA aqueous extract compared to extracts made with other solvents (87, 88). It would be interesting to compare the composition of extracts made using traditional preparation methods to CAW. In the US, as in Asian countries (81, 89, 90), CA is a popular component of herbal teas prepared by extracting dried CA herb with hot water. This extraction process closely mirrors the preparation of CAW and would be expected to yield similar components, although comparative dosing would need to be evaluated.

## Data Availability Statement

The original contributions presented in the study are included in the article/Supplementary Material, further inquiries can be directed to the corresponding author/s.

## Author Contributions

KMW, AAM, JR, DK, JFS, CSM, JFQ, and AS participated in research design. KMW, AAM, JM, MC, DK, and AS conducted the experiments and performed data analysis. KMW, DK, and AS wrote or contributed to the writing of the manuscript. KMW, JFQ, and AS secured funding. All authors contributed to the article and approved the submitted version.

## Funding

This work was supported by NIH-NCCIH grants: R61AT009628, T32AT002688; NIH-NIA pilot grant: P30AG008017; NIHNCRR grants: S10RR027878, S10RR025628; and Department of Veterans Affairs Merit Review Grant IO1BX003440.

## Conflict of Interest

AS is an *ad hoc* consultant for Oregon's Wild Harvest. Services provided by Ashland Laboratories and Oregon's Wild Harvest were financed by the NIH grant R61AT009628 awarded to Oregon Health & Science University. Co-authors affiliated with Oregon's Wild Harvest (JM and JR) contributed to product design, manufacture and quality control of CAP. The CAP product and placebo described in this study were made for research purposes only and not for commercial use. The remaining authors declare that the research was conducted in the absence of any commercial or financial relationships that could be construed as a potential conflict of interest.

## Publisher's Note

All claims expressed in this article are solely those of the authors and do not necessarily represent those of their affiliated organizations, or those of the publisher, the editors and the reviewers. Any product that may be evaluated in this article, or claim that may be made by its manufacturer, is not guaranteed or endorsed by the publisher.
