# Supplementary material for: Developing a Rational, Optimized Product of Centella asiatica for Examination in Clinical Trials: Real World Challenges
Source: Front Nutr. 2022 Jan 14;8:799137. doi: 10.3389/fnut.2021.799137 (PMC8797052; doi:10.3389/fnut.2021.799137)
Supplement: Supplementary file 1 [file Data_Sheet_1.PDF]

Table S1. Results of microbial testing of *Centella asiatica* botanical raw material samples evaluated for the manufacture of a *Centella asiatica* clinical trial product (CAP), Gotu kola preblend manufacturing intermediate and final products CAP 0g, CAP 2g and CAP 4 g.

CA-1 plant material (trade sample)

Test	Supplier's data	OWH data	OWH Specifications
**Coliforms**	N/A	<10 cfu/g	Report only
**E.coli**	Absent	<10 cfu/g /not detected	<10 mpn/g /not detected
**Salmonella**	Absent	Absent in 25 g	Absent in 25 g
**APC**	7885 cfu/g	4,800 cfu/g	<10,000,000 cfu/g
**Mold**	Yeast+mold 854 cfu/g	<100 cfu/g	<100,000 cfu/g
**Yeast**	Yeast+mold 854 cfu/g	2,400 cfu/g	<100,000 cfu/g

CA-2 plant material (trade sample)

Test	Supplier's data	OWH data	OWH Specifications
**Coliforms**	N/A	N/A	Report only
**E.coli**	N/A	Conforms	<10 mpn/g /not detected
**Salmonella**	N/A	Conforms	Absent in 25 g
**APC**	N/A	N/A	<10,000,000 cfu/g
**Mold**	N/A	N/A	<100,000 cfu/g
**Yeast**	N/A	N/A	<100,000 cfu/g

CA-3 plant material (trade sample)

Test	Supplier's data	OWH data	OWH Specifications
**Coliforms**	<100 cfu/g	<10 cfu/g	Report only
**E.coli**	Negative	<10 cfu/g /not detected	<10 mpn/g /not detected
**Salmonella**	Negative	Negative	Absent in 25 g
**APC**	12,500 cfu/g	200,700 cfu/g	<10,000,000 cfu/g
**Mold**	Yeast and mold 450 cfu/g	<500 cfu/g	<100,000 cfu/g
**Yeast**	Yeast and mold 450 cfu/g	600 cfu/g	<100,000 cfu/g

CA-3 plant material (bulk sample)

Test	Supplier's data	OWH data	OWH Specifications
**Coliforms**	<100 cfu/g	<10 cfu/g	Report only
**E.coli**	Negative	<10 cfu/g	<10 mpn/g /not detected
**Salmonella**	Negative	Negative	Absent in 25 g
**APC**	12,500 cfu/g	<5000 cfu/g	<10,000,000 cfu/g
**Mold**	Yeast and mold 450 cfu/g	<500 cfu/g	<100,000 cfu/g
**Yeast**	Yeast and mold 450 cfu/g	500 cfu/g	<100,000 cfu/g

CA-6 plant material (trade sample)

Test	Supplier's data	OWH data	OWH Specifications
**Coliforms**	100 cfu/g	N/A	Report only
**E.coli**	Absent/10g	N/A	<10 mpn/g /not detected
**Salmonella**	Negative/25g	N/A	Absent in 25 g
**APC**	50,000 cfu/g	N/A	<10,000,000 cfu/g
**Mold**	100 cfu/g	N/A	<100,000 cfu/g
**Yeast**	100 cfu/g	N/A	<100,000 cfu/g

CA-6 plant material (bulk sample)

Test	Supplier's data	OWH data	OWH Specifications
**Coliforms**	100 cfu/g	>7000 cfu/g	Report only
**E.coli**	Absent/10g	<10 cfu/g	<10 mpn/g /not detected
**Salmonella**	Negative/25g	Negative	Absent in 25 g
**APC**	50,000 cfu/g	10,000 cfu/g	<10,000,000 cfu/g
**Mold**	100 cfu/g	<500 cfu/g	<100,000 cfu/g
**Yeast**	100 cfu/g	<500 cfu/g	<100,000 cfu/g

CA-7 plant material (trade sample)

Test	Supplier's data	OWH data	OWH Specifications
**Coliforms**	<10 cfu/g	N/A	Report only
**E.coli**	<10 cfu/g	N/A	<10 mpn/g /not detected
**Salmonella**	Negative	N/A	Absent in 25 g
**APC**	<100 cfu/g	N/A	<10,000,000 cfu/g
**Mold**	<100 cfu/g	N/A	<100,000 cfu/g
**Yeast**	<100 cfu/g	N/A	<100,000 cfu/g

CA-8 plant material (trade sample)

Test	Supplier's data	OWH data	OWH Specifications
**Coliforms**	<3 mpn/g	N/A	Report only
**E.coli**	<3 mpn/g	N/A	<10 mpn/g /not detected
**Salmonella**	Negative/25g	N/A	Absent in 25 g
**APC**	380 cfu/g	N/A	<10,000,000 cfu/g
**Mold**	<10 cfu/g	N/A	<100,000 cfu/g
**Yeast**	<10 cfu/g	N/A	<100,000 cfu/g

Gotu kola preblend (CAW spray dried extract blended from two batches)

Test	OWH data	OWH Specifications
**Coliforms**	<10 cfu/g	Report only
**E.coli**	<10 mpn/g	<10 mpn/g /not detected
**Salmonella**	Negative	Absent in 25 g
**APC**	N/A	<10,000,000 cfu/g
**Mold**	N/A	<100,000 cfu/g
**Yeast**	N/A	<100,000 cfu/g

CAP 0g

Test	OWH data	OWH Specifications
**Coliforms**	<10 cfu/g	Report only
**E.coli**	<10 mpn/g	<10 mpn/g /not detected
**Salmonella**	Negative	Absent in 25 g
**APC**	<500 cfu/g	<10,000,000 cfu/g
**Mold**	<500 cfu/g	<100,000 cfu/g
**Yeast**	<500 cfu/g	<100,000 cfu/g

CAP 2g

Test	OWH data	OWH Specifications
**Coliforms**	<10 cfu/g	Report only
**E.coli**	<10 mpn/g	<10 mpn/g /not detected
**Salmonella**	Negative	Absent in 25 g
**APC**	<5000 cfu/g	<10,000,000 cfu/g
**Mold**	<10,000 cfu/g	<100,000 cfu/g
**Yeast**	<500 cfu/g	<100,000 cfu/g

CAP 4g

Test	OWH data	OWH Specifications
**Coliforms**	<10 cfu/g	Report only
**E.coli**	<10 mpn/g	<10 mpn/g /not detected
**Salmonella**	Negative	Absent in 25 g
**APC**	<500 cfu/g	<10,000,000 cfu/g
**Mold**	<500 cfu/g	<100,000 cfu/g
**Yeast**	<500 cfu/g	<100,000 cfu/g

Suppliers data were taken from certificates of analysis; OWH = Oregon's Wild Harvest (Redmond, OR); "OWH data" were obtained by microbial testing in Oregon's Wild Harvest quality control laboratories; N/A = not available (data either not provided or not measured); APC = aerobic plate count; cfu = colony forming units; mpn = most probable number.
